# Lipometabolism and Glycometabolism in Liver Diseases

**DOI:** 10.1155/2018/1287127

**Published:** 2018-12-12

**Authors:** Hao-ran Ding, Jing-lin Wang, Hao-zhen Ren, Xiao-lei Shi

**Affiliations:** ^1^Department of Hepatobiliary Surgery, Nanjing Drum Tower Hospital Clinical College of Nanjing Medical University, Nanjing, China; ^2^Department of Hepatobiliary Surgery, The Affiliated Drum Tower Hospital of Nanjing University Medical School, Nanjing, China

## Abstract

The liver is the main metabolic organ in the body especially in lipometabolism and glycometabolism. Carbohydrates and fats disorders can result in insulin resistance in the liver. Metabolic imbalance can even lead to life-threatening conditions. Therefore, it is essential to maintain the normal metabolic function of the liver. When the liver is in a pathological state, liver metabolism homeostasis is damaged, and metabolic disorders will further aggravate liver disease. Consequently, it is essential to determine the relationship between liver diseases and metabolic disorders. Here we review a lot of evidence that liver diseases are closely related to lipometabolism and glycometabolism. Although the disorder of the liver metabolism is caused by different liver diseases, the break of metabolic balance is determined by changes in the state of the liver. We discuss the relationship between liver disease and metabolic changes, outline the process of how metabolic changes are regulated by liver diseases, and describe the role which metabolic changes play in the process and prognosis of liver disease.

## 1. Introduction

The liver is the largest organ in the body and mainly regulates carbohydrate and lipid metabolism. The abnormal metabolism of carbohydrates and fats due to an imbalance in hepatic metabolism can result in insulin resistance in insulin sensitive tissues such as the liver. An imbalance in hepatic metabolism may result from the disease which causes hepatic dysfunction. Several metabolites of carbohydrates and fats can even lead to life-threatening conditions. As a consequence, it is essential to maintain the normal metabolic function of the liver.

## 2. Metabolism of Major Substances in the Liver

### 2.1. Lipometabolism

An important function of the liver is lipid metabolism. Lipid intake, esterification, oxidation, and the secretion of fatty acids take place in hepatocytes. Triglycerides are delivered to the liver for lipid metabolism and absorbed by liver cells, which is regulated by LDL (low density lipoprotein) receptors and LRP (LDL receptor-related proteins) [1, 2]. Excess carbohydrates can be transformed into lipids in the liver under the regulation of transcription factors such as SREBP1, ChREBP, and LXR which is referred to as the de novo fatty acid synthesis pathway [3].

When hepatic lipid metabolism homeostasis is damaged, triglycerides are accumulated pathologically in liver cells due to upregulation of triglyceride synthesis, decreased lipid droplet decomposition, and impaired triglyceride and very LDL (VLDL) secretory function [4]. Dysregulation of hepatic lipid metabolism homeostasis will ultimately result in fatty liver. The mechanism involved in the progression of nonalcoholic fatty liver disease (NAFLD) to nonalcoholic steatohepatitis (NASH), liver fibrosis, liver cirrhosis, and liver cancer is still unclear. Ekstedt M and Angulo P found that fibrosis contributed to the development of chronic liver disease in patients with NAFLD [5, 6]. Although liver fibrosis is due to excessive activation of hepatic stellate cells (HSCs), the process is regulated by lipid metabolism [7, 8]. Therefore, it is crucial to maintain normal lipid metabolism homeostasis for the healthy biological function of the liver.

### 2.2. Glycometabolism

The liver also plays a significant role in sugar metabolism, which is responsible for the formation and storage of glucose. Following food consumption, glucose metabolism in the liver results in a rapid transformation from glucose synthesis to glucose storage and is regulated by insulin, a key regulator [9]. Insulin contributes to glucose storage by activating glycogen synthase which mediates the synthesis of hepatic glycogen. When the secretion of insulin is insufficient, the synthesis of hepatic glycogen is inhibited. This is illustrated by hepatic glycogen synthesis in patients with type 1 diabetes and a normal diet, which is only 1/3 of that in healthy individuals [10]. The biological function of insulin is dependent on coordinating intracellular signaling pathways. Insulin can activate IRTK (insulin receptor tyrosine kinase), which mediates the phosphorylation of ATP to stimulate the metabolism of glucose in association with PDK1 and mTORC2. Insulin can also downregulate glycogen related enzymes and inactivate glycogen synthase kinase which reduces the production of glucose in the liver.

### 2.3. The Relationship between Glucose Metabolism and Lipid Metabolism

Sugar metabolism is closely related to lipid metabolism. Insulin resistance is usually accompanied by liver steatosis. When insulin resistance occurs, lipolysis is suppressed and lipid synthesis is increased due to the effect of hyperinsulinemia. Abnormal lipid metabolism, especially the accumulation of ectopic lipids, is closely associated with insulin resistance. Samuel VT found that the inhibitory effect of insulin on liver gluconeogenesis was significantly reduced [11]. Diacylglycerol is a product of lipolysis, which can activate PKC to impair the insulin signal. Raddatz K found that although the fat content in the liver increased, the level of insulin resistance decreased significantly in high fat fed Prkce (a core gene which promotes lipolysis to upregulate diacylglycerol content) knockout mice [12]. Ectopic lipid accumulation in the liver can activate related pathways which regulate insulin function resulting in reduced glucose uptake and hepatic glycogen synthesis in vivo. The accumulation of ectopic lipid increases the transport of glucose to the liver and stimulates de novo lipid synthesis which can lead to hyperlipidemia. In addition, in a metabolic and inflammatory environment caused by lipid accumulation, macrophages are increased in white adipose tissue which promotes the esterification of fatty acids and induces hyperlipidemia. Macrophages can also regulate lipolysis, which results in the transportation of fatty acids to the liver and the accumulation of acetyl-CoA in the liver. Acetyl-CoA is a strong activator of pyruvate carboxylase, which can promote the transformation of glycerol to glucose known as gluconeogenesis [13].

When the liver is in a pathological state, liver metabolism homeostasis is damaged, and metabolic disorders will further aggravate liver disease. Consequently, it is essential to determine the relationship between liver diseases and metabolic disorders.

## 3. Major Liver Diseases

### 3.1. Hepatic Steatosis

Hepatic steatosis is one of the major diseases affecting human health worldwide. The NAFLD morbidity rate is approximately 25% [14]. When liver cell damage, inflammation, and fibrosis occur, simple hepatic steatosis is transformed into NASH which is a high risk factor for the development of hepatocellular carcinoma (HCC) [15]. When lipid metabolism is abnormal, the balance of lipid composition in cells is disrupted. Due to the pathological accumulation of lipids, the development of lipotoxicity results in dysfunction of organelles, which leads to cell dysfunction and even death. Signal recognition and transduction in cells depend on normal lipid metabolism. Lipids present in cell membranes and the cytoplasm can be directly modified by cell kinases to regulate cell behavior [16]. Triglycerides are the main components of lipid droplets, and if the content of triglycerides in lipid droplets is high or low, this can lead to lipid metabolism disorders. Papazyan R found that when the synthesis of triglycerides was reduced by depressing diacylglycerol acylase, oxidative stress, inflammation, fibrosis, and cell damage were aggravated despite a reduction in hepatic steatosis [17]. Perilipin-5 regulates the storage of triglycerides in lipid droplets. If the expression of perilipin-5 is downregulated, this leads to pathological lipolysis and lipotoxicity although the lipid droplets are small [18]. Consequently, it is beneficial for health that triglycerides are stored in an inert storage mode to prevent the development of NASH from NAFLD. The study by Raichur S showed an interesting phenomenon, where a significant increase in ceramide content was observed in a mouse model of NASH [19]. Ceramide is an intermediate metabolite of sphingolipid which can be upregulated by high expression of proinflammatory factors. Ceramide can accelerate the progression of hepatic steatosis to NASH due to the promotion of inflammation by interacting with TNF-*α*. When the expression of ceramide is downregulated, complications such as fatty liver, cell damage, and insulin resistance in the NASH model can be alleviated.

Lipotoxicity not only is harmful to cells under the regulation of inflammatory mechanisms, endoplasmic reticulum stress, and reactive oxygen species (ROS), but also affects the biological functions of organelles. The most important organelles affected are mitochondria and the endoplasmic reticulum [20]. Studies have shown that increasing the level of autophagy in the liver in the nonalcoholic fatty liver model can eliminate polarization and damaged mitochondria [21]. This process reduces ATP consumption and the production of free radicals which is caused by the reverse function of mitochondrial ATP synthetase. In contrast, if mitochondrial autophagy is suppressed, severe liver mitochondrial damage and hepatic steatosis will develop [22]. Therefore, mitochondrial autophagy plays an important role in the reduction of hepatic steatosis and inhibition of the progression of NAFLD to NASH. In addition to mitochondrial autophagy, liver steatosis can also cause the changes of mitochondrial dynamics. Excessive fat accumulation can directly lead to the mitochondrial damage [23]. Nakagawa H found that endoplasmic reticulum stress plays an important role in the development of NASH from hepatic steatosis. When the level of endoplasmic reticulum stress was improved in mice, obvious NASH characteristics appeared in hepatocytes, such as balloon degeneration, inflammatory infiltration, and bridging “netted” fibrosis. When hepatic steatosis has progressed to NASH, it can quickly develop into a liver adenoma and eventually lead to HCC [24].

In addition to lipotoxicity, “glucose toxicity” caused by glucose metabolism disorders is involved in the pathogenesis of NASH. Excess carbohydrates activate the fat synthesis pathway which is regulated by acetyl-CoA carboxylase, SCD-1, and fatty acid synthase to aggravate hepatic steatosis. Fructose can increase the expression of CD36 and is associated with de novo lipid synthesis-related proteins such as ChREBP to promote lipid synthesis [25]. Excess glucose and fructose can regulate the expression of ChREBP and SREBP1c directly. In addition, fructose can also improve the downstream fat synthesis gene to promote the accumulation of lipids. Softic S confirmed that hepatic steatosis in mice was aggravated following the inhibition of fructose metabolism. The incidence of hepatic steatosis was also significantly increased in humans with fructose metabolism disorders [26].

### 3.2. Hepatic Fibrosis

Most chronic liver injuries can evolve into liver fibrosis which is closely related to the activation of HSCs. The process by which HSCs are activated involves the transformation of HSCs from the static state in Vitamin A lipid droplets to the facilitated fibrous state [27]. The HSCs in the facilitated fibrous state can transdifferentiate into myofibroblasts and produce superfluous ECM (extracellular matrix) which is responsible for liver fibrosis. In this process, the intracellular vitamin A will be lost. Liver fibrosis is an effective indicator of liver-related complications and specific liver diseases. Liver disease can lead to vitamin A homeostasis impaired and lead to vitamin A deficiency eventually. Lipolysis can stimulate the activation of HSCs which can promote the secretion of extracellular matrix and accelerate the process of liver fibrosis [28]. Hepatic steatosis in the long term may evolve to liver fibrosis. Cholesterol is a confirmed endogenous activator of LXR (liver X receptor) which can not only regulate lipometabolism and glycometabolism but also modulate the activation of HSCs [29]. In addition to lowering cholesterol uptake, LXR prevents the progression of liver fibrosis. Beaven, S. W et al. found that the mice which lack LXR*α* and LXR*β* are easier to develop liver fibrosis [30]. It has been proved that lipid droplet-associated protein RAB18 plays an important role in the metabolism of vitamins and cholesterol [31]. Lipid metabolism disorder can lead to the downregulation of PPAR and/or the dysfunction of PPAR. PPAR can reverse the process of HSCs activation and prevent the development of liver fibrosis by downregulating the expression of fibrogenic cytokines secreted by HSCs. Studies have shown that the inhibition of PPAR can increase the risk of suffering liver fibrosis and the activation of PPAR can reduce liver injury and liver fibrosis [32, 33]. It is strange that the expression of BMP6 (bone morphogenetic protein) is upregulated in NAFLD but not in other liver diseases. In the study in vitro, it is found that BMP6 inhibits the activity of HSCs and reduces the expression of the fibrotic gene [34]. There must be a connection between lipid metabolism and BMP6 activation, but the specific mechanism is not clear. Macrophages play an important role in the progression of liver fibrosis because it has obvious improvement of liver fibrosis after exhaustion of macrophages [35, 36]. During metabolic disorder, CCL2 and MCP-1 can activate fibroblast-related macrophages such as phagocytes, Kupffer cells, and macrophages induced by monocytes which result in upregulated metabolic inflammation [37]. This process of cell recruitment aggravates the chronic inflammatory reaction in the liver. Mononuclear cells can secrete fibrotic factors such as TGF-*β* and PDGF [38]. These fibrotic factors not only upregulate monocyte-induced macrophages, but also promote the differentiation of HSCs into myofibroblasts. The above process is the main source of extracellular matrix, especially collagen, in liver fibrosis caused by chronic inflammation [39]. The IL-1 and TNF pathways can prolong the survival time of HSCs which is regulated by mononuclear cells [36]. Kazankov K analyzed macrophage markers in more than 300 patients with hepatic steatosis. He found that the level of macrophage activation is closely related to the grade of liver fibrosis and disease [40]. Lipid metabolism also participates in the activation of macrophages [41]. Adipocytokines play multiple roles in the progression from NAFLD to liver cirrhosis. Leptin promotes the liver fibrosis, but adiponectin prevents the progress of liver fibrosis [42]. Leptin can upregulate the expression of the *α*SMA, Type 1 Collagen, and TGF*β*. Hedgehog pathway which is closely associated with liver fibrosis is regulated by lipid [43]. The Hedgehog pathway can activate HSCs to transform it into proliferating myofibroblast. When NAFLD progresses to NASH whose pathological feature is liver fibrosis, the expression of Hedgehog is significantly upregulated [44]. Hedgehog signaling pathway is also involved in lipid synthesis and sugar utilization [45]. Guy CD found that when the Hedgehog signaling pathway is suppressed, the accumulation of muscle fibroblasts in the liver can be reduced [46].

### 3.3. Hepatocellular Carcinoma

Although the main risk factors for HCC are HBV and HCV infection, HCC induced by virus infection is decreasing annually due to the development of effective hepatitis B vaccines and anti-HCV drugs [47, 48]. Obesity, a high risk factor for HCC, has attracted significant attention [49]. When lipid metabolism is disturbed, the balance is broken between lipid synthesis and lipid decomposition. The decrease of lipoprotein synthesis and lipophagy can result in lipotoxicity which may cause chronic liver injury [50]. Lipotoxicity is one of the important mechanisms for the development of NASH to HCC. Consequently, the metabolic related factors such as type II diabetes, obesity, and metabolic syndrome are becoming the risk factors of HCC. Scientists are paying more and more attention to metabolic disorders and HCC. People with abdominal obesity have a higher risk of HCC [51]. Visceral fat is of great significance in predicting the recurrence of HCC [52]. The lipid accumulation caused by abnormal lipid metabolism may result in the remodeling of adipose tissue. In the reconstituted adipose tissue, the differentiation of the adipocytes is activated and the angiogenesis appears, which provides a favorable microenvironment for the development of HCC. Not only that, abnormal lipid metabolism can lead to the disordered secretory of adipokine characterized by leptin reduction and increased adiponectin. Leptin can not only promote the development of liver fibrosis, but also promote the development of HCC by activating JAK/STAT pathway and ERK pathway. Upregulated leptin can inhibit the apoptosis of tumor tissue through suppressing antiapoptotic pathway mediated by TGF*β* [53]. Leptin plays a role in promoting the proliferation of tumor cells. What is more, leptin can also provide a microenvironment for tumor growth. The biological function of adiponectin is opposite to leptin. Adiponectin can inhibit the proliferation of tumor cells through regulating AMPK signaling pathway and mTOR signaling pathway. Jiang CM found that low adiponectin is closely related to the occurrence of HCC [54]. Senescent adipocytes which secret more cytokines can aggravate chronic inflammation associated with obesity. The process above which is called the SASP can promote the development of HCC. When the SASP process is suppressed, the incidence of HCC is significantly reduced [55]. Chronic inflammation caused by liver metabolic diseases, liver fibrosis, the death of parenchymal cells, and lack of monitoring the proliferation of abnormal liver parenchymal cells are the main risk factors for HCC. Park EJ conducted research on the correlation between obesity and liver cancer, which confirmed that there is a relationship between abnormal lipid metabolism and the development of HCC. The authors found that male mice with a high fat diet were more likely to develop HCC. This may be related to the activation of HSCs and hepatocytes, which can secrete TNF and IL-6 to activate STAT3. STAT3 is an important tumor transcription factor, which can promote the occurrence of liver cancer [56]. In addition to the theory of inflammatory induction, some scholars believe that the progression of NASH to HCC is caused by the TNF signal activated by TNFR1. The TNFR1 signal in hepatocytes and hepatoma progenitor cells can be upregulated by IKK*β* to activate the NF-*κ*B signaling pathway which mediates cell proliferation. Nakagawa H found that inhibition of the TNFR1 signal not only prevented the progression of NAFLD to NASH, but also decreased the incidence of HCC [24]. With abnormal lipid metabolism, the production of ROS is increased by upregulating *β*-oxidation and *ω*-oxidation. Oxidative stress can damage the genome and aggravate the mitochondrial burden. The injured liver cells can also activate the JNK pathway which is related to cell survival and stress. Metabolic disorder of lipids also plays an important role in tumor recurrence [57]. A prospective study found that excessive ROS caused by abnormal lipid metabolism can increase the risk of recurrence after liver cancer surgery [58].

Besides lipid metabolism, the occurrence of HCC is closely related to glucose metabolism. Weng C J and Li C et al. carried out a follow-up analysis of IGF-1 (insulin growth factor 1) and HbA1C (glycosylated hemoglobin A1C) in diabetic patients and found that blood glucose and hyperinsulinemia were significantly related to the incidence of HCC. Effective blood glucose control can reduce the degree of chronic liver disease and reduce the incidence of HCC [59]. Hyperinsulinemia can promote the development of liver fibrosis by activating HSCs and promoting angiogenesis. Continuous hyperinsulinemia can promote the development of liver cancer by modulating IGF signaling axis which can promote the proliferation of hepatocytes and angiogenesis [60]. The upregulated insulin and IGF can bind to the insulin receptor and promote the formation of tumors under the interaction of IGFR. This may be related to the P13K/Akt pathway and MAPK pathway. Increased blood glucose and insulin increase the level of IGF-1 and its bioavailability. Activated IGF-1 can inhibit the receptor-mediated apoptosis pathway in tumor cells and promote their proliferation [61]. Hyperglycemia and insulin resistance increase the expression of proinflammatory factors TNF-*α* and IL-6, which can lead to chronic inflammation. Hyperglycemia can induce the accumulation of ROS. Excess ROS can be combined with DNA which leads to the development of HCC [62, 63].

### 3.4. Acute Liver Failure

Acute liver failure is characterized by massive liver cell necrosis. Acute liver injury is often accompanied by metabolic disorders, acidosis, and sepsis. These complications can lead to hypoglycemia, hypokalemia, lactic acidosis, and hyperammonemia. Abnormal levels of metabolites can induce coma. Metabolic dysfunction is an important factor in the deterioration of acute liver failure [64]. Abnormality of lipoprotein metabolism is caused by acute liver failure and increases the content of cholesterol on the cell membrane. This process can reduce the deformability of cells. This is the reason that cells are more vulnerable to damage in people with abnormal lipid metabolism. Lysosomal lipase is an important enzyme which can hydrolyze triglycerides and cholesterol. This enzyme is essential to decompose lipid into free cholesterol and free fatty acids. When this enzyme is suppressed, lipid can deposit in the liver and finally form into acute liver failure [65]. The disease which lacks lysosomal lipase is called Wolman disease (lysosomal acid lipase deficiency). Abnormal glycometabolism also induces acute liver failure. When the people are short of hepatic fructose 1-phosphate aldolase, eating fructose can lead to acute liver failure. Acute liver failure can result in OS (oxidative stress), which brings about changes in mitochondrial structural proteins and DNA resulting in ATP depletion and impaired ATP production. Excess accumulation of ROS can induce a shift in JNK proteins in mitochondria, which are responsible for mitochondrial inactivation, aggravation of OS, and liver injury [66]. There is transient lipid accumulation in the liver after major hepatectomy. Therefore, acute liver failure after hepatectomy is often accompanied by lipid metabolism disorders. Ekaterina Kachaylo found that improving liver lipid metabolism can reduce liver failure after hepatectomy by reducing lipid accumulation in the liver [67]. Correction of the metabolic disorder in acute liver failure is the key to improving the prognosis of patients. The timely correction of abnormal biochemical indicators can reduce the impact of metabolic disorders on the body.

## 4. Conclusions

The liver is an important metabolic organ. Liver abnormalities affect metabolic homeostasis, and metabolites can in turn play a protective or aggravating role in the diseased liver. In view of the complex relationship between the liver and metabolism, there is great potential in the treatment of specific liver diseases with targeted metabolic therapy. This treatment is expected to delay or even cure the disease and has considerable clinical value.

## Abbreviations

LDL: Low density lipoprotein
LRP: LDL receptor-related proteins
SREBP1: Sterol Regulatory Element Binding Proteins 1
ChREBP: Carbohydrate response element binding protein
LXR: Liver X receptor
VLDL: Very low density lipoprotein
NAFLD: Nonalcoholic fatty liver disease
NASH: Nonalcoholic steatohepatitis
HSCs: Hepatic stellate cells
IRTK: Insulin receptor tyrosine kinase
ATP: Adenosine Triphosphate
PDK1: Phosphoinositide-dependent kinase 1
mTORC2: mTOR Complex 2
PKC: Protein kinase C
HCC: Hepatocellular carcinoma
TNF-*α*: Tumor necrosis factor-*α*
ROS: Reactive oxygen species
SCD-1: Stearoyl-CoA desaturase 1
CD36: Fatty acid translocase
SREBP1c: Sterol Regulatory Element Binding Proteins 1c
HSCs: Hepatic stellate cells
ECM: Extracellular matrix
RAB18: Ras-related protein Rab-18
PPAR: Peroxisome proliferator-activated receptor
BMP6: Bone morphogenetic protein 6
CCL2: Chemokine CCL2
MCP-1: Monocyte chemoattractant protein-1
TGF-*β*: Transforming growth factor-*β*
PDGF: Platelet-derived growth factor
IL-1: Interleukin-1
TNF: Tumor necrosis factor
*α*SMA: *α* smooth muscle actin
HBV: Hepatitis B virus
HCV: Hepatitis C virus
JAK: Janus kinase 1
STAT: Signal Transducers and Activators of Transcription
ERK: Extracellular Signal Regulated Kinase
AMPK: Adenosine Monophosphate Activated Protein Kinase
mTOR: Mammalian target of rapamycin
SASP: Senescence-associated secretory phenotype
IL-6: Interleukin-6
TNFR1: Tumor Necrosis Factor Receptor 1
IKK*β*: I*κ*B kinase *β*
NF-*κ*B: Nuclear factor-*κ*B
IGF-1: Insulin growth factor 1
HbA1C: Glycosylated hemoglobin A1C
IGFR: Insulin-Like Growth Factor Receptor
JNK: c-Jun N-terminal kinase
OS: Oxidative stress.

## References

[1] Rohlmann A., Gotthardt M., Hammer R. E., Herz J. (1998). Inducible inactivation of hepatic LRP gene by Cre-mediated recombination confirms role of LRP in clearance of chylomicron remnants. *The Journal of Clinical Investigation*.

[2] Hussain M. M., Maxfield F. R., Más-Oliva J., Tabas I., Ji Z. S., Innerarity T. L. (1991). Clearance of chylomicron remnants by the low density lipoprotein receptor-related protein/alpha 2-macroglobulin receptor. *The Journal of Biological Chemistry*.

[3] Wang Y., Viscarra J., Kim S.-J., Sul H. S. (2015). Transcriptional regulation of hepatic lipogenesis. *Nature Reviews Molecular Cell Biology*.

[4] Kawano Y., Cohen D. E. (2013). Mechanisms of hepatic triglyceride accumulation in non-alcoholic fatty liver disease. *Journal of Gastroenterology*.

[5] Ekstedt M., Hagström H., Nasr P. (2015). Fibrosis stage is the strongest predictor for disease-specific mortality in NAFLD after up to 33 years of follow-up. *Hepatology*.

[6] Angulo P., Kleiner D. E., Dam-Larsen S. (2015). Liver fibrosis, but no other histologic features, is associated with long-term outcomes of patients with nonalcoholic fatty liver disease. *Gastroenterology*.

[7] Chen M., Liu J., Yang W., Ling W. (2017). Lipopolysaccharide mediates hepatic stellate cell activation by regulating autophagy and retinoic acid signaling. *Autophagy*.

[8] Lu C., Xu W., Zheng S. (2017). Nrf2 activation is required for curcumin to induce lipocyte phenotype in hepatic stellate cells. *Biomedicine & Pharmacotherapy*.

[9] Petersen K. F., Laurent D., Rothman D. L., Cline G. W., Shulman G. I. (1998). Mechanism by which glucose and insulin inhibit net hepatic glycogenolysis in humans. *The Journal of Clinical Investigation*.

[10] Hwang J.-H., Perseghin G., Rothman D. L. (1995). Impaired net hepatic glycogen synthesis in insulin-dependent diabetic subjects during mixed meal ingestion: A 13C nuclear magnetic resonance spectroscopy study. *The Journal of Clinical Investigation*.

[11] Samuel V. T., Liu Z.-X., Qu X. (2004). Mechanism of hepatic insulin resistance in non-alcoholic fatty liver disease. *The Journal of Biological Chemistry*.

[12] Raddatz K., Turner N., Frangioudakis G. (2011). Time-dependent effects of Prkce deletion on glucose homeostasis and hepatic lipid metabolism on dietary lipid oversupply in mice. *Diabetologia*.

[13] Samuel V. T., Shulman G. I. (2016). The pathogenesis of insulin resistance: integrating signaling pathways and substrate flux. *The Journal of Clinical Investigation*.

[14] Satapathy S. K., Sanyal A. J. (2015). Epidemiology and Natural History of Nonalcoholic Fatty Liver Disease. *Seminars in Liver Disease*.

[15] Rinella M. E. (2015). Nonalcoholic fatty liver disease: a systematic review. *The Journal of the American Medical Association*.

[16] Perry R. J., Zhang X. M., Zhang D., Kumashiro N., Camporez J. P., Cline G. W. (2014). Leptin reverses diabetes by suppression of the hypothalamic-pituitary-adrenal axis. *NAT MED*.

[17] Papazyan R., Sun Z., Kim Y. H. (2016). Physiological Suppression of Lipotoxic Liver Damage by Complementary Actions of HDAC3 and SCAP/SREBP. *Cell Metabolism*.

[18] Wang C., Zhao Y., Gao X. (2015). Perilipin 5 improves hepatic lipotoxicity by inhibiting lipolysis. *Hepatology*.

[19] Raichur S., Wang S., Chan P. (2014). CerS2 Haploinsufficiency Inhibits *β*-Oxidation and Confers Susceptibility to Diet-Induced Steatohepatitis and Insulin Resistance. *Cell Metabolism*.

[20] Ertunc M. E., Hotamisligil G. S. (2016). Lipid signaling and lipotoxicity in metaflammation: indications for metabolic disease pathogenesis and treatment. *Journal of Lipid Research*.

[21] Wang L., Liu X., Nie J. (2015). ALCAT1 controls mitochondrial etiology of fatty liver diseases, linking defective mitophagy to steatosis. *Hepatology*.

[22] Williams J. A., Ni H., Ding Y., Ding W. (2015). Parkin regulates mitophagy and mitochondrial function to protect against alcohol-induced liver injury and steatosis in mice. *American Journal of Physiology-Gastrointestinal and Liver Physiology*.

[23] Ipsen D. H., Lykkesfeldt J., Tveden-Nyborg P. (2018). Molecular mechanisms of hepatic lipid accumulation in non-alcoholic fatty liver disease. *Cellular and Molecular Life Sciences*.

[24] Nakagawa H., Umemura A., Taniguchi K. (2014). ER Stress Cooperates with Hypernutrition to Trigger TNF-Dependent Spontaneous HCC Development. *Cancer Cell*.

[25] Ter Horst K. W., Serlie M. J. (2017). Fructose consumption, lipogenesis, and non-alcoholic fatty liver disease. *Nutrients*.

[26] Softic S., Gupta M. K., Wang G.-X. (2017). Divergent effects of glucose and fructose on hepatic lipogenesis and insulin signaling. *The Journal of Clinical Investigation*.

[27] Puche J. E., Saiman Y., Friedman S. L. (2013). Hepatic stellate cells and liver fibrosis. *Comprehensive Physiology*.

[28] Friedman S. L. (2008). Hepatic stellate cells: protean, multifunctional, and enigmatic cells of the liver. *Physiological Reviews*.

[29] Tsuchida T., Friedman S. L. (2017). Mechanisms of hepatic stellate cell activation. *Nature Reviews Gastroenterology & Hepatology*.

[30] Beaven S. W., Wroblewski K., Wang J. (2011). Liver X receptor signaling is a determinant of stellate cell activation and susceptibility to fibrotic liver disease. *Gastroenterology*.

[31] O'Mahony F., Wroblewski K., O'Byrne S. M. (2015). Liver X receptors balance lipid stores in hepatic stellate cells through Rab18, a retinoid responsive lipid droplet protein. *Hepatology*.

[32] Morán-Salvador E., Titos E., Rius B. (2013). Cell-specific PPAR*γ* deficiency establishes anti-inflammatory and anti-fibrogenic properties for this nuclear receptor in non-parenchymal liver cells. *Journal of Hepatology*.

[33] Staels B., Rubenstrunk A., Noel B. (2013). Hepatoprotective effects of the dual peroxisome proliferator-activated receptor alpha/delta agonist, GFT505, in rodent models of nonalcoholic fatty liver disease/nonalcoholic steatohepatitis. *Hepatology*.

[34] Arndt S., Wacker E., Dorn C. (2015). Enhanced expression of BMP6 inhibits hepatic fibrosis in non-alcoholic fatty liver disease. *Gut*.

[35] Sunami Y., Leithäuser F., Gul S. (2012). Hepatic activation of IKK/NF*κ*B signaling induces liver fibrosis via macrophage-mediated chronic inflammation. *Hepatology*.

[36] Pradere J., Kluwe J., de Minicis S. (2013). Hepatic macrophages but not dendritic cells contribute to liver fibrosis by promoting the survival of activated hepatic stellate cells in mice. *Hepatology*.

[37] Krenkel O., Tacke F. (2017). Liver macrophages in tissue homeostasis and disease. *Nature Reviews Immunology*.

[38] Mossanen J. C., Krenkel O., Ergen C. (2016). Chemokine (C-C motif) receptor 2–positive monocytes aggravate the early phase of acetaminophen-induced acute liver injury. *Hepatology*.

[39] Weiskirchen R., Tacke F. (2016). Liver fibrosis: from pathogenesis to novel therapies. *Digestive Diseases*.

[40] Kazankov K., Barrera F., Møller H. J. (2016). The macrophage activation marker sCD163 is associated with morphological disease stages in patients with non-alcoholic fatty liver disease. *Liver International*.

[41] Dennis E. A., Deems R. A., Harkewicz R. (2010). A mouse macrophage lipidome. *The Journal of Biological Chemistry*.

[42] Saxena N. K., Anania F. A. (2015). Adipocytokines and hepatic fibrosis. *Trends in Endocrinology & Metabolism*.

[43] Myers B., Sever N., Chong Y. (2013). Hedgehog pathway modulation by multiple lipid binding sites on the smoothened effector of signal response. *Developmental Cell*.

[44] Syn W.-K., Jung Y., Omenetti A. (2009). Hedgehog-mediated epithelial-to-mesenchymal transition and fibrogenic repair in nonalcoholic fatty liver disease. *Gastroenterology*.

[45] Teperino R., Amann S., Bayer M. (2012). Hedgehog partial agonism drives warburg-like metabolism in muscle and brown fat. *Cell*.

[46] Guy C. D., Suzuki A., Abdelmalek M. F., Burchette J. L., Diehl A. M. (2015). Treatment response in the PIVENS trial is associated with decreased hedgehog pathway activity. *Hepatology*.

[47] El-Serag H. B. (2012). Epidemiology of viral hepatitis and hepatocellular carcinoma. *Gastroenterology*.

[48] Kimer N., Dahl E. K., Gluud L. L., Krag A. (2012). Antiviral therapy for prevention of hepatocellular carcinoma in chronic hepatitis C: Systematic review and meta-analysis of randomised controlled trials. *BMJ Open*.

[49] Sun B., Karin M. Obesity, inflammation, and liver cancer.

[50] (2016). Hepatocellular Carcinoma in Non-alcoholic Fatty Liver Disease: Epidemiology, Pathogenesis, and Prevention. *Journal of Clinical and Translational Hepatology*.

[51] Karagozian R., Derdák Z., Baffy G. (2014). Obesity-associated mechanisms of hepatocarcinogenesis. *Metabolism*.

[52] Lo G.-H. (2010). Is visceral fat accumulation really an independent risk factor for hepatocellular carcinoma recurrence after curative treatment in patients with suspected NASH?. *Gut*.

[53] Shetty K., Chen J., Shin J., Jogunoori W., Mishra L. (2015). Pathogenesis of hepatocellular carcinoma development in non-alcoholic fatty liver disease. *Current Hepatology Reports*.

[54] Jiang C.-M., Pu C.-W., Hou Y.-H., Chen Z., Alanazy M., Hebbard L. (2014). Non alcoholic steatohepatitis a precursor for hepatocellular carcinoma development. *World Journal of Gastroenterology*.

[55] Yoshimoto S., Tze Mun L., Koji A., Hiroaki K., Seidai S., Seiichi O. (2013). *Obesity-induced gut microbial metabolite promotes liver cancer through senescence secretome*.

[56] He G., Yu G. Y., Temkin V., Ogata H., Kuntzen C., Sakurai T. (2010). *Hepatocyte IKKbeta/NF-kappaB inhibits tumor promotion and progression by preventing oxidative stress-driven STAT3 activation*.

[57] Fei W.-y., Wu R.-q., Zhou S.-f. (2001). Optimal consumption choices with anticipation: methods of martingale. *Journal of Mathematics*.

[58] Suzuki Y., Imai K., Takai K. (2013). Hepatocellular carcinoma patients with increased oxidative stress levels are prone to recurrence after curative treatment: A prospective case series study using the d-ROM test. *Journal of Cancer Research and Clinical Oncology*.

[59] Weng C., Hsieh Y., Tsai C. (2010). Relationship of Insulin-Like Growth Factors System Gene Polymorphisms with the Susceptibility and Pathological Development of Hepatocellular Carcinoma. *Annals of Surgical Oncology*.

[60] Siddique A., Kowdley K. V. (2011). Insulin Resistance and Other Metabolic Risk Factors in the Pathogenesis of Hepatocellular Carcinoma. *Clinics in Liver Disease*.

[61] Newschaffer C. J. (2002). Premenopausal Levels of Circulating Insulin-Like Growth Factor I and the Risk of Post-Menopausal Breast Cancer: A Population-Based, Nested Case-Control Study. *Defense Technical Information Center*.

[62] Tanaka H., Fujita N., Sugimoto R. (2008). Hepatic oxidative DNA damage is associated with increased risk for hepatocellular carcinoma in chronic hepatitis C. *British Journal of Cancer*.

[63] Leahy J. (2007). Activation of Oxidative Stress by Acute Glucose Fluctuations Compared With Sustained Chronic Hyperglycemia in Patients With Type 2 Diabetes. *Yearbook of Endocrinology*.

[64] Bernal W., Auzinger G., Dhawan A., Wendon J. (2010). Acute liver failure. *The Lancet*.

[65] Pericleous M., Kelly C., Wang T., Livingstone C., Ala A. (2017). Wolman's disease and cholesteryl ester storage disorder: the phenotypic spectrum of lysosomal acid lipase deficiency. *The Lancet Gastroenterology and Hepatology*.

[66] Ye D., Wang Y., Li H. (2014). Fibroblast growth factor 21 protects against acetaminophen-induced hepatotoxicity by potentiating peroxisome proliferator-activated receptor coactivator protein-1*α*-mediated antioxidant capacity in mice. *Hepatology*.

[67] Kachaylo E., Tschuor C., Calo N. (2017). PTEN Down-Regulation Promotes *β*-Oxidation to Fuel Hypertrophic Liver Growth After Hepatectomy in Mice. *Hepatology*.

